# Clinical Outcomes of Pembrolizumab in Head and Neck Squamous Cell Carcinoma Subsites Excluded from the KEYNOTE-048 Trial

**DOI:** 10.3390/curroncol33010057

**Published:** 2026-01-18

**Authors:** Gai Yamashita, Takuro Okada, Isaku Okamoto, Takahito Kondo, Tatsuya Ito, Shota Fujii, Takuma Kishida, Yusuke Aihara, Kenji Hanyu, Yuri Ueda, Kunihiko Tokashiki, Hiroki Sato, Yuki Harada, Kaho Momiyama, Takashi Matsuki, Yukiomi Kushihashi, Tatsuo Masubuchi, Yuichiro Tada, Taku Yamashita, Kiyoaki Tsukahara

**Affiliations:** 1 Department of Otorhinolaryngology, Head and Neck Surgery, Tokyo Medical University, Tokyo 160-0023, Japan; gny-1021@tokyo-med.ac.jp (G.Y.); tsuka@tokyo-med.ac.jp (K.T.); 2 Department of Otorhinolaryngology, Head and Neck Surgery, Tokyo Medical University Hachioji Medical Center, Tokyo 193-0998, Japan; 3 Department of Otorhinolaryngology, Head and Neck Surgery, Kitasato University School of Medicine, Sagamihara 252-0374, Japan; 4 Department of Head and Neck Oncology and Surgery, International University of Health and Welfare Mita Hospital, Tokyo 108-8329, Japan

**Keywords:** pembrolizumab, recurrent or metastatic squamous cell carcinoma of the head and neck, KEYNOTE-048, excluded subsites

## Abstract

Recurrent or metastatic squamous cell carcinoma of the head and neck (R/M SCCHN) carries a poor prognosis; however, immune checkpoint inhibitors have emerged as critical therapeutic options. Although the KEYNOTE-048 trial established the efficacy of pembrolizumab, the population was restricted to major sites (e.g., oral cavity, oropharynx, hypopharynx, and larynx), excluding subsites such as the paranasal sinuses and nasopharynx. To evaluate outcomes in these populations, we conducted a multicenter retrospective study of 167 patients with R/M SCCHN treated with pembrolizumab between December 2019 and February 2022. The cohort comprised 127 patients with tumors in included sites and 27 in excluded subsites. Primary endpoints included overall survival (OS), progression-free survival (PFS), overall response rate (ORR), disease control rate (DCR), and immune-related adverse events (irAEs). In the excluded subsite group, median OS was 15.2 months (1-year rate: 70.6%), and median PFS was 4.9 months (1-year rate: 21.2%). The ORR was 22.2% and the DCR was 59.3%. The incidence of irAEs was 25.9%, with Grade ≥ 3 events in 3.7%. Survival outcomes did not differ significantly from those in included sites. These findings suggest the potential efficacy and safety of pembrolizumab in subsites excluded from KEYNOTE-048, warranting validation in prospective trials.

## Simple Summary

Recurrent or metastatic squamous cell carcinoma of the head and neck (R/M SCCHN) carries a poor prognosis. Although the KEYNOTE-048 trial established the efficacy of pembrolizumab, it was restricted to major sites such as the oral cavity, oropharynx, hypopharynx, and larynx. Subsites including the paranasal sinuses, nasopharynx, external ear, and salivary glands were excluded, and evidence regarding these locations is lacking. In this multicenter retrospective study, we evaluated 167 patients treated with pembrolizumab, including 27 with tumors in excluded subsites. The median overall survival in the excluded subsite group was 15.2 months (1-year rate: 70.6%), and median progression-free survival was 4.9 months (1-year rate: 21.2%). Survival outcomes in this group did not differ significantly from those in included sites. The safety profile was manageable. This study is the first to demonstrate the potential efficacy and safety of pembrolizumab in patients with tumors in subsites excluded from KEYNOTE-048.

## 1. Introduction

The prognosis for patients with recurrent or metastatic squamous cell carcinoma of the head and neck (R/M SCCHN) remains poor. The median overall survival (mOS) is approximately 6 months [1,2], and the 1-year mortality rate approaches 90% following the diagnosis of recurrence or metastasis [3].

In the international phase III CheckMate-141 trial, the PD-1 monoclonal antibody nivolumab significantly prolonged OS compared with investigator’s choice chemotherapy in patients with platinum-refractory R/M SCCHN [4]. Furthermore, the international phase III KEYNOTE-048 trial established the efficacy of the PD-1 monoclonal antibody pembrolizumab in patients with platinum-naïve or platinum-sensitive R/M SCCHN [5]. When pembrolizumab monotherapy or pembrolizumab combined with chemotherapy was compared with cetuximab plus platinum and 5-fluorouracil (the standard treatment group), both pembrolizumab arms demonstrated a significant improvement in OS. Consequently, nivolumab is recommended in Japan for platinum-refractory R/M SCCHN, whereas pembrolizumab is recommended for platinum-naïve or platinum-sensitive R/M SCCHN. Similarly, the National Comprehensive Cancer Network (NCCN) guidelines in the United States list nivolumab and pembrolizumab as Category 1 recommended agents for recurrent or metastatic SCCHN [6].

However, the KEYNOTE-048 trial applied strict eligibility criteria regarding primary tumor location, allowing enrollment only for tumors arising in the oral cavity, oropharynx, hypopharynx, and larynx [5].

Several anatomical subsites—such as the paranasal sinuses, nasopharynx, external ear, and salivary glands—were intentionally excluded because these malignancies differ substantially in epidemiology, viral association, molecular biology, and standard treatment strategies [6,7,8].

For example, nasopharyngeal carcinoma is strongly associated with Epstein–Barr virus (EBV) infection and exhibits a distinct immune microenvironment compared with conventional SCCHN [7], whereas sinonasal carcinomas demonstrate unique molecular, pathological, and clinical characteristics [8].

Similarly, salivary gland carcinomas encompass diverse histologic subtypes with heterogeneous biological behavior [9], and external ear carcinomas are rare tumors with distinct management strategies [10].

Recent studies have begun to evaluate immune checkpoint inhibitors in these excluded subsites. In nasopharyngeal carcinoma, the phase III KEYNOTE-122 trial compared pembrolizumab with chemotherapy but did not demonstrate a survival advantage, although pembrolizumab showed a favorable safety profile [11]. For salivary gland carcinoma, pembrolizumab demonstrated modest activity in the KEYNOTE-028 and KEYNOTE-158 studies, with durable responses observed in a subset of patients [12,13]. Additionally, retrospective analyses suggest that immune checkpoint inhibitors may offer clinical benefit in sinonasal squamous cell carcinoma, although evidence remains limited [14].

Despite these emerging data, no study has directly evaluated pembrolizumab in a real-world cohort of patients with tumors arising from subsites excluded from KEYNOTE-048. Therefore, the clinical efficacy and safety of pembrolizumab in these populations remain unclear. Moreover, the rarity of these excluded subsites has historically limited the ability to conduct prospective trials, resulting in a fragmented evidence base composed largely of small, single-institution studies. Differences in tumor biology, treatment accessibility, and diagnostic pathways further complicate the extrapolation of data from conventional SCCHN to these unique anatomical locations. As immune checkpoint inhibitors continue to expand across tumor types, clarifying their role in these understudied subsites is essential for optimizing treatment selection and improving patient outcomes in real-world clinical practice.

To address this gap, we conducted a multicenter retrospective study to evaluate outcomes of pembrolizumab in patients with tumors located in subsites excluded from KEYNOTE-048 and compared them with outcomes in patients with tumors arising from included sites.

## 2. Materials and Methods

### 2.1. Patients

We retrospectively reviewed the medical records of patients treated between December 2019 and February 2022 at four institutions (Tokyo Medical University Hospital, Tokyo Medical University Hachioji Medical Center, International University of Health and Welfare Mita Hospital, and Kitasato University Hospital). Because this study used existing clinical information only and involved no direct patient contact or interventions, the requirement for individual informed consent was waived. Information about the study was disclosed on each hospital’s website, and patients were provided the opportunity to opt out. The study was approved by the institutional review boards of the participating hospitals (approval nos. T2022-0048, 5-22-3, C22-007) and was conducted in accordance with the Declaration of Helsinki. In addition to demographic and clinical characteristics, detailed information regarding prior treatments, comorbidities, and baseline laboratory data was collected when available. Baseline imaging studies were reviewed to confirm the primary tumor site and the presence of measurable lesions according to RECIST version 1.1 [15]. Patients were included regardless of whether they received pembrolizumab monotherapy or pembrolizumab in combination with chemotherapy, reflecting real-world clinical practice. Patients with insufficient imaging data, unclear primary tumor origin, or non-squamous histology were excluded to ensure diagnostic accuracy and consistency across institutions.

### 2.2. Treatment and Follow-Up

Patients received pembrolizumab at a dose of 200 mg/body every 3 weeks or 400 mg/body every 6 weeks. Cisplatin was administered at a dose of 80 mg/m^2^ every 3 weeks, while 5-fluorouracil was delivered at 800 mg/m^2^ for 4 consecutive days on the same cycle schedule. For patients with impaired renal function or a prior history of Grade ≥3 toxicities, cisplatin was replaced with carboplatin. Carboplatin was given at an area-under-the-curve target of 4 mg/mL·min every 3 weeks. Up to six cycles of pembrolizumab combined with FP were provided, after which pembrolizumab monotherapy was continued as maintenance therapy. Follow-up evaluations included physical examinations, laboratory tests, and imaging studies. Target lesions were reassessed every 2–3 months with either computed tomography or magnetic resonance imaging. Additional imaging, such as positron emission tomography or ultrasound, was performed at the discretion of the treating physician when clinically indicated. Treatment continuation was determined based on radiologic or clinical disease progression, the development of intolerable adverse events, or physician judgment. Patients who discontinued pembrolizumab were followed until death or the last known clinical encounter to ensure accurate survival assessment.

### 2.3. Endpoint

The study endpoints consisted of overall survival (OS), progression-free survival (PFS), overall response rate (ORR; defined as the sum of complete and partial responses), disease control rate (DCR; defined as complete response, partial response, or stable disease), and the frequency of immune-related adverse events (irAEs). OS was defined as the interval from the initiation of pembrolizumab to death or the final follow-up. PFS was defined as the interval from the initiation of pembrolizumab to disease progression or death. ORR was evaluated according to RECIST version 1.1 [15], whereas irAEs were graded using the Common Terminology Criteria for Adverse Events version 5.0 [16].

ORR and DCR analyses were restricted to patients with evaluable lesions on imaging. Patients with clinically apparent disease progression were classified as having progressive disease. Eastern Cooperative Oncology Group PS was assessed prior to the initiation of pembrolizumab. OS and PFS were stratified by the presence of irAEs and the best overall response (BOR), respectively.

Tumor response assessments were performed by radiologists or experienced head and neck surgeons at each institution. When discrepancies occurred, consensus was reached through multidisciplinary review. Management of irAEs, including corticosteroid use, treatment interruption, or permanent discontinuation, was recorded when available.

### 2.4. Statistical Analysis

Survival outcomes were analyzed using the Kaplan–Meier method, and differences between groups were assessed with the log-rank test. Hazard ratios were derived from the Cox proportional hazards model. A *p*-value of <0.05 was considered statistically significant. Comparisons of ORR, DCR, and the occurrence of irAEs were conducted using Fisher’s exact test.

All statistical analyses were carried out with EZR (Saitama Medical Center, Jichi Medical University, Saitama, Japan), a graphical interface for R (R Foundation for Statistical Computing, Vienna, Austria) [17].

To ensure consistency across institutions, all survival and response data were centrally reviewed, and discrepancies in event dates or response classification were resolved through consensus among the investigators. Baseline variables were checked for completeness before analysis, and data entry accuracy was verified by cross-referencing clinical records. All analyses were performed using standardized procedures to maintain reproducibility and minimize potential bias in outcome assessment.

## 3. Results

### 3.1. Patient Cohort

**Figure 1:**
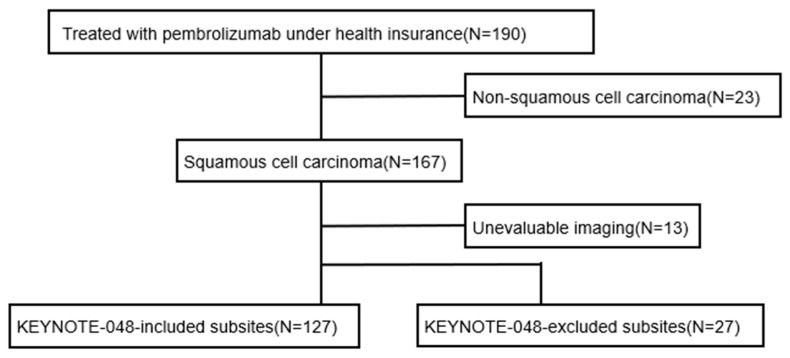
Flowchart outlining the selection of study subjects.

A total of 190 patients who received pembrolizumab under the national health insurance system during the study period were evaluated. Histological analysis excluded 23 patients with non-squamous cell carcinoma, leaving 167 patients with squamous cell carcinoma (Figure 1). Thirteen patients were excluded due to a lack of evaluable imaging data. Patients with primary tumors located in sites included in the KEYNOTE-048 trial (oral cavity, oropharynx, hypopharynx, and larynx) were defined as the “included site” group (*n* = 127). Patients with primary tumors located in sites not included in the KEYNOTE-048 trial were defined as the “excluded subsite” group (*n* = 27).

In addition to these exclusions, we reviewed the clinical records to confirm that all remaining patients had adequate baseline evaluations, including laboratory tests and imaging studies, to ensure accurate assessment of treatment outcomes. Among the 167 eligible patients, the majority had undergone prior locoregional or systemic therapy before initiating pembrolizumab, reflecting real-world treatment patterns in Japan. The distribution of primary tumor sites and exclusion criteria was consistent across the participating institutions, suggesting minimal institutional bias in patient selection.

### 3.2. Patient Characteristics

The baseline characteristics of the included site group (*n* = 127) are summarized in Table 1. Approximately 95% of patients had a performance status (PS) of 0–1, although a small minority with PS 2–3 received treatment. Men comprised 86.6% of the group. The most common primary sites were the hypopharynx (44.9%), followed by the oral cavity (23.6%), oropharynx (23.6%), and larynx (7.9%). The median age was 71 years (range, 34–89 years). A combined positive score (CPS) ≥ 20 was observed in 63.0% of patients, and 78.0% received pembrolizumab monotherapy.

The baseline characteristics of the excluded subsite group (*n* = 27) are summarized in Table 2. Approximately 89% had a PS of 0–1. Men comprised 59.3% and women 40.7% of the cohort. The primary tumor sites included the paranasal sinuses (37.0%), nasopharynx (29.6%), external ear (14.8%), and salivary glands (11.1%). The median age was 65 years (range, 45–89 years). A CPS ≥ 20 was observed in 55.6% of patients, and 92.6% received pembrolizumab monotherapy.

### 3.3. Treatment Efficacy

**Figure 2:**
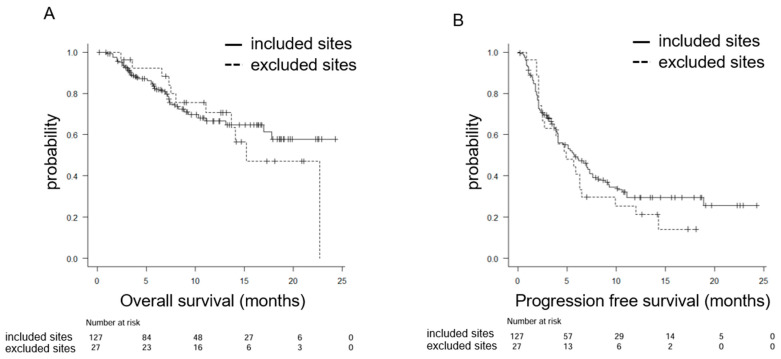
Kaplan–Meier plots for (**A**) overall survival (OS) and (**B**) progression-free survival (PFS) classified by anatomical location: included sites and excluded subsites, according to the eligibility criteria of the KEYNOTE-048 trial.

Figure 2 illustrates the Kaplan–Meier survival curves. In the included site group, the mOS was not reached (95% CI: 17–NA), and the 1-year OS rate was 66.5% (Figure 2A). The median progression-free survival (mPFS) was 5.8 months (95% CI: 4.1–7.6), with a 1-year PFS rate of 29.4% (Figure 2B).

In the excluded subsite group, the mOS was 15.2 months (95% CI: 11.1–NA), with a 1-year OS rate of 70.6% (Figure 2A). The mPFS was 4.9 months (95% CI: 2.4–6.5), with a 1-year PFS rate of 21.2% (Figure 2B). No statistically significant differences were observed between the two groups regarding mOS (*p* = 0.726) or mPFS (*p* = 0.383).

Treatment responses are summarized in Table 3. In the included site group, the BOR distribution was as follows: CR in 11.8%, PR in 24.4%, SD in 26.0%, and PD in 37.8%. This yielded an ORR of 36.2% and a DCR of 62.2%. In the excluded subsite group, CR was observed in 3.7%, PR in 18.5%, SD in 37.0%, and PD in 40.7%; the ORR was 22.2% and the DCR was 59.3%. The differences in ORR (*p* = 0.19) and DCR (*p* = 0.52) between the groups were not statistically significant.

### 3.4. Safety

We also evaluated the safety profile. In the included site group, the incidence of irAEs was 18.1%, compared with 25.9% in the excluded subsite group. This difference was not statistically significant (*p* = 0.42) (Table 3).

In the included site group, the most common irAEs were erythema multiforme (2.4%), hypothyroidism (2.4%), interstitial lung disease (2.4%), colitis (1.6%), and adrenal insufficiency (1.6%); Grade ≥ 3 irAEs occurred in 5.6% of patients (Table 4). In the excluded subsite group, the most common irAEs included erythema multiforme (11.1%), colitis (3.7%), adrenal insufficiency (3.7%), iritis (3.7%), and hypothyroidism (3.7%); Grade ≥ 3 irAEs occurred in 3.7% of patients (Table 5).

Most irAEs were manageable and resolved with appropriate supportive care, including corticosteroid administration and temporary treatment interruption when necessary. No treatment-related deaths occurred in either group. The slightly higher incidence of erythema multiforme in the excluded subsite group may reflect site-specific immune responses, particularly in tumors arising from chronically inflamed mucosal surfaces such as the paranasal sinuses or nasopharynx. Although the overall frequency of Grade ≥3 events was low, clinicians should remain vigilant for immune-related toxicities even in rare anatomical subsites, where atypical presentations may occur. Importantly, the absence of unexpected or severe irAEs in the excluded subsite group supports the feasibility of pembrolizumab in these populations. Further research is warranted to explore whether tumor location or associated viral status (e.g., EBV in nasopharyngeal carcinoma) influences the spectrum or severity of irAEs. Prospective studies incorporating immune profiling and longitudinal toxicity monitoring may help clarify these associations and guide personalized management strategies.

## 4. Discussion

This study is the first to report the clinical outcomes of pembrolizumab in patients with tumors arising from subsites excluded from the KEYNOTE-048 trial.

Although the KEYNOTE-048 trial established pembrolizumab as a standard first-line therapy for R/M SCCHN, its eligibility criteria were deliberately limited to tumors of the oral cavity, oropharynx, hypopharynx, and larynx [5]. As a result, malignancies of the paranasal sinuses, nasopharynx, external ear, and salivary glands were not represented in the pivotal trial. These exclusions were based on the distinct epidemiological, viral, molecular, and therapeutic characteristics of these tumors [6,7,8,9,10], rather than on evidence suggesting a lack of benefit from pembrolizumab. The design of KEYNOTE-048 inherently limited the generalizability of its findings to these anatomical subsites. Because the same eligibility criteria were applied to both the pembrolizumab and chemotherapy arms, tumors arising from these excluded locations were not included in any study group [5]. As a result, no direct comparison between pembrolizumab and standard chemotherapy exists for these rare anatomical locations, underscoring the need for real-world evidence.

In the KEYNOTE-048 trial, the mOS in the pembrolizumab monotherapy arm was approximately 13.0 months, and the mPFS was 2.3 months [5]. In real-world studies, Fan et al. reported an mOS of 15.97 months and an mPFS of 8.53 months in patients with R/M SCCHN treated with pembrolizumab [18], whereas Okada et al. reported an mOS of 22.7 months and an mPFS of 5.1 months in the pembrolizumab monotherapy group [19].

In our analysis, the included site group demonstrated an mOS that was not reached (95% CI: 17–NA), a 1-year OS rate of 66.5%, an mPFS of 5.8 months (95% CI: 4.1–7.6), and a 1-year PFS rate of 29.4%. Although direct comparisons are challenging, our findings appear broadly consistent with those of the KEYNOTE-048 trial and other real-world studies.

In the excluded subsite group, the mOS was 15.2 months (95% CI: 11.1–NA), the 1-year OS rate was 70.6%, the mPFS was 4.9 months (95% CI: 2.4–6.5), and the 1-year PFS rate was 21.2%. No statistically significant differences were observed when compared with the included site group. These findings suggest that pembrolizumab may provide clinical benefit even in patients with tumors arising from subsites excluded from the KEYNOTE-048 trial.

Beyond the clinical outcomes observed in this study, the heterogeneity of tumors arising from excluded subsites warrants further consideration. These malignancies encompass diverse biological backgrounds, including viral-associated carcinogenesis in nasopharyngeal carcinoma [20,21], high-grade transformation and molecular diversity in salivary gland tumors [22], and chronic inflammatory or environmental exposures in sinonasal cancers [23]. Such variability may influence both tumor immunogenicity and responsiveness to PD-1 blockade. Moreover, differences in diagnostic pathways and treatment accessibility across subsites could contribute to variations in disease burden at the time of pembrolizumab initiation. As immune checkpoint inhibitors continue to expand across tumor types, understanding how anatomical and biological context shapes therapeutic benefit will be increasingly important. Integrating molecular profiling, immune microenvironment analysis [24], and real-world treatment patterns may help identify subgroups most likely to benefit from pembrolizumab. These insights could ultimately guide more personalized treatment strategies for patients with rare head and neck cancer subsites.

Several prior studies have evaluated pembrolizumab or other immune checkpoint inhibitors in these excluded subsites. In nasopharyngeal carcinoma, the phase III KEYNOTE-122 trial did not show a survival advantage for pembrolizumab over chemotherapy, although durable responses were observed and the safety profile was favorable [11]. For salivary gland carcinomas, pembrolizumab demonstrated modest but clinically meaningful activity in the KEYNOTE-028 and KEYNOTE-158 trials [12,13]. Retrospective studies of sinonasal squamous cell carcinoma have reported that immune checkpoint inhibition may be a viable option for these rare tumors [14]. Although direct comparisons across studies are limited, the consistency of response patterns supports the biological plausibility of pembrolizumab efficacy in these subsites.

The incidence of irAEs was 18.1% (23/127) in the included site group and 25.9% (7/27) in the excluded subsite group; the difference between the two groups was not statistically significant (*p* = 0.42). The most common irAEs in the included site group were hypothyroidism (2.4%), erythema multiforme (2.4%), interstitial lung disease (2.4%), colitis (1.6%), and adrenal insufficiency (1.6%); Grade ≥ 3 events occurred in 5.6% (7/127) of patients. In the excluded subsite group, erythema multiforme (11.1%), colitis (3.7%), adrenal insufficiency (3.7%), iritis (3.7%), and hypothyroidism (3.7%) were observed, with Grade ≥ 3 events occurring in 3.7% (1/27) of patients. In the KEYNOTE-048 trial, irAEs occurred in 17% of patients [5]. Real-world studies have reported higher incidences of irAEs, ranging from 40% to 50% [18,25]. Our study adds to this body of evidence by providing a unified analysis of multiple excluded subsites within a single cohort. Notably, the safety profile in the excluded subsite group was comparable to that in included sites and to previously published data, with no unexpected toxicities. This is clinically relevant given concerns about immune-related toxicity in tumors with unique microenvironments, such as EBV-associated nasopharyngeal carcinoma or chronically inflamed sinonasal tumors [26,27,28].

Another clinical challenge associated with excluded subsites is the lack of standardized chemotherapy regimens. The NCCN Head and Neck Cancers guidelines treat nasopharyngeal carcinoma, salivary gland tumors, and sinonasal tumors as separate entities, providing site-specific recommendations rather than a unified regimen for excluded subsites [6].

## 5. Conclusions

In conclusion, this study is the first to demonstrate the efficacy and safety of pembrolizumab in patients with tumors in subsites excluded from the KEYNOTE-048 trial. For malignancies arising in anatomical locations with limited treatment options, the potential role of immune checkpoint inhibitors is clinically meaningful. However, the retrospective design and relatively small sample size—particularly within each subsite category—limit the statistical power to detect subtle differences. Additionally, the absence of a chemotherapy control group for excluded subsites prevents direct comparison with standard regimens. Even so, given the rarity of these tumors and the lack of prospective data, our findings provide valuable real-world evidence supporting the potential role of pembrolizumab in these understudied populations. Future prospective studies or pooled analyses will be essential to validate these observations.

## Figures and Tables

**Table 1:** Baseline clinical characteristics of patients with included sites.

Clinical Characteristics			
Factor	Group	*N* = 127	%
ECOG performance status (%)	0	83	65.4
	1	37	29.1
	2	6	4.7
	3	1	0.8
Gender (%)	Women	17	13.4
	Men	110	86.6
Platinum (%)	No	93	73.2
	Sensitive	28	22.0
	Unfit	6	4.7
UICC stage (%)	Stage I/II/III	43	33.9
	Stage IV	84	66.1
Age	Median	71	55.9
	Range	34–89	
Pembrolizumab	Pembrolizumab monotherapy	99	78.0
	Pembrolizumab with chemotherapy	28	22.0
**Pathological/Biological Characteristics**			
**Factor**	**Group**	***N* = 127**	**%**
Primary site (%)	Oral cavity	30	23.6
	Oropharynx	30	23.6
	p16+	20	15.7
	p16−	8	6.3
	Unknown	2	1.6
	Hypopharynx	57	44.9
	Larynx	10	7.9
CPS	≥20	80	63.0
	1~19	45	35.4
	<1	0	0.0
	Unknown	2	1.6

CPS: Combined positive score.

**Table 2:** Baseline clinical characteristics of patients with excluded subsites.

Clinical Characteristics			
Factor	Group	*N* = 27	%
ECOG performance status (%)	0	14	51.9
	1	10	37.0
	2	3	11.1
Gender (%)	Women	11	40.7
	Men	16	59.3
Platinum (%)	No	1	3.7
	Sensitive	9	33.3
	Unfit	17	63.0
UICC Stage (%)	Stage I/II/III	11	40.7
	Stage IV	16	59.3
Age	Median	65	
	Range	45–89	
Pembrolizumab	Pembrolizumab monotherapy	25	92.6
	Pembrolizumab with chemotherapy	2	7.4
**Pathological/Biological Characteristics**			
**Factor**	**Group**	***N* = 27**	**%**
Primary site (%)	Nasopharynx	8	29.6
	Paranasal sinus	10	37.0
	Salivary glands	3	11.1
	External ear	4	14.8
	Unknown	2	7.4
CPS	≥20	15	55.6
	1~19	7	25.9
	<1	2	7.4
	Unknown	3	11.1

**Table 3:** Tumor response and immune-related adverse events in included vs. excluded subsites.

	Included Sites		Excluded Subsites		
	*N* = 127	%	*N* = 27	%	*p*-Value
Best overall response					
Complete response (CR)	15	11.8	1	3.7	
Partial response (PR)	31	24.4	5	18.5	
Stable disease (SD)	33	26.0	10	37.0	
Progressive disease (PD)	48	37.8	11	40.7	
Overall response rate (ORR)	46	36.2	6	22.2	0.19
Disease control rate (DCR)	79	62.2	16	59.3	0.52
Immune-related adverse events (irAE)					
No	104	81.9	20	74.1	
Yes	23	18.1	7	25.9	0.42

**Table 4:** Summary of adverse events (included sites).

	All Grades		Grade 3, 4	
	*N*	%	*N*	%
Erythema multiforme	3	2.4	0	0.0
Colitis	2	1.6	2	1.6
Adrenal insufficiency	2	1.6	0	0.0
Hypothyroidism	3	2.4	0	0.0
Liver dysfunction	1	0.8	0	0.0
Bullous pemphigoid	1	0.8	0	0.0
Rheumatoid arthritis	1	0.8	0	0.0
Pancreatitis	1	0.8	1	0.8
Stomatitis	1	0.8	0	0.0
Infusion reaction	1	0.8	0	0.0
Mucositis	1	0.8	1	0.8
Interstitial lung disease	3	2.4	0	0.0
Myocarditis	1	0.8	1	0.8
Diabetes	1	0.8	1	0.8
Electrolyte abnormality	1	0.8	0	0.0
Hypophysitis	1	0.8	1	0.8

**Table 5:** Summary of adverse events (excluded subsites).

	All Grades		Grade 3, 4	
	*N*	%	*N*	%
Erythema multiforme	3	11.1	0	0
Colitis	1	3.7	1	3.7
Adrenal insufficiency	1	3.7	0	0
Iritis	1	3.7	0	0
Hypothyroidism	1	3.7	0	0

## Data Availability

The data presented in this study are available on request from the corresponding author due to hospital policy.

## Abbreviations

The following abbreviations are used in this manuscript:

AUC	Area under the curve
BOR	Best overall response
CPS	Combined positive core
CR	Complete response
DCR	Disease control rate
irAE	Immune-related adverse event
mOS	Median overall survival
mPFS	Median progression-free survival
NCCN	National Comprehensive Cancer Network
ORR	Overall response rate
OS	Overall survival
PD	Progression disease
PFS	Progression-free survival
PR	Partial response
PS	Performance status
R/M SCCHN	Recurrent or metastatic squamous cell carcinoma of the head and neck
SD	Stable disease
